# Efficiency optimization in quantum computing: balancing thermodynamics and computational performance

**DOI:** 10.1038/s41598-024-55314-z

**Published:** 2024-02-24

**Authors:** Tomasz Śmierzchalski, Zakaria Mzaouali, Sebastian Deffner, Bartłomiej Gardas

**Affiliations:** 1 Institute of Theoretical and Applied Informatics, Polish Academy of Sciences, Bałtycka 5, 44-100 Gliwice, Poland; 2 Department of Physics, University of Maryland, Baltimore County, Baltimore, MD 21250 USA; 3 National Quantum Laboratory, College Park, MD 20740 USA

**Keywords:** Quantum thermodynamics, Quantum annealing, Quantum computation, Quantum information, Thermodynamics

## Abstract

We investigate the computational efficiency and thermodynamic cost of the D-Wave quantum annealer under reverse-annealing with and without pausing. Our demonstration on the D-Wave 2000*Q* annealer shows that the combination of reverse-annealing and pausing leads to improved computational efficiency while minimizing the thermodynamic cost compared to reverse-annealing alone. Moreover, we find that the magnetic field has a positive impact on the performance of the quantum annealer during reverse-annealing but becomes detrimental when pausing is involved. Our results, which are reproducible, provide strategies for optimizing the performance and energy consumption of quantum annealing systems employing reverse-annealing protocols.

## Introduction

Large-scale investments in quantum technologies are usually justified with promised advantages in sensing, communication, and computing^1^. Among these, quantum computing is probably the most prominent application since it has the potential to revolutionize information processing and computational capabilities. For certain tasks, quantum computers exploit the fundamental principles of quantum mechanics to perform complex calculations exponentially faster than classical computers^2–4^. The tremendous computational power offered by quantum systems has fueled excitement and exploration in various scientific, industrial, and financial sectors^1,5–12^. Consequently, there have been significant developments in the pursuit of quantum advantage that have propelled quantum computing from theoretical speculation to practical implementation^13–23^.

Major technology companies such as IBM, Google, Microsoft, Intel, and Nvidia have been investing massively in quantum research and development, leading to the establishment of quantum computing platforms and open-source frameworks, that enable researchers and developers to demonstrate and explore the potential of quantum algorithms and applications^24^. These advancements have been driven by breakthroughs in both hardware and algorithmic techniques, bringing us closer to realizing the potential of quantum computers^25,26^.

However, the rapid development of quantum technologies also raises critical questions about the energy requirements and environmental implications of quantum computation^27,28^. Energy consumption has become a focal point for researchers, policymakers, and society at large as the demand for computing power continues to rise, and concerns about climate change and sustainability intensify. Consequently, assessing the energy consumption of quantum computers is vital for evaluating their feasibility, scalability, and identifying potential bottlenecks^29^.

The energy consumption of quantum computers stems from various sources, including the cooling systems needed to maintain the delicate quantum states, the control and manipulation of qubits, and the complex infrastructure required to support quantum operations^30,31^. The superposition characteristic of qubits demands a sophisticated physical environment, with precise temperature control and isolation, leading to significant energy expenditures. These challenges call for synergic work between quantum information science, quantum engineering, and quantum physics to develop an interdisciplinary approach to tackle this problem^32^.

The theoretical framework to quantify the energy consumption of quantum computation is through quantum thermodynamics, which provides the necessary tools to quantify and characterize the efficiency of emerging quantum technologies, and therefore is crucial in laying a roadmap to scalable devices^27,33,34^. The field of quantum thermodynamics provides a theoretical foundation to understand the fundamental limits of quantum technologies, macroscopically, in terms of energy consumption and efficiency, by identifying the thermodynamic resources required to process and manipulate quantum information at the microscopic level^27,35,36^, such as quantum versions of Landauer’s principle^37–41^. The exploration of the thermodynamics of information is not limited to the equilibrium settings, as recent research has delved into the nonequilibrium aspects of quantum computation, particularly in the context of quantum algorithms and quantum simulation^42,43^. Understanding the thermodynamics of quantum systems, including the generation of entropy, heat dissipation, and non-equilibrium dynamics, serves to optimize the algorithmic performance, energy consumption, and resource utilization^44^.

The study of the thermodynamics of quantum computers has been an active research area with notable results that deepen our understanding of the energy landscapes, heat dissipation, and efficiency of quantum computation while also addressing challenges related to noise, decoherence, and thermal effects^45–48^. The optimization of the energy efficiency of quantum computers has been approached from several angles, for instance, to eluciate the minimization of the energy dissipation during computations, and to develop energy-efficient algorithms and architectures^49–57^. The focus is on reducing energy requirements and increasing the computational efficiency of quantum systems, paving the way for sustainable and practical quantum computing technologies. As quantum computers generate heat during operation, effective thermal management becomes essential to maintain qubit stability and mitigate thermal errors.

In this paper, we study the interplay between thermodynamic and computational efficiency in quantum annealing. In recent years, thermodynamic considerations of the D-Wave quantum annealer have become prevalent. For instance, some of us used the quantum fluctuation theorem to assess the performance of annealing^48^. Furthermore, the working mechanism of the D-Wave chip was shown to be equivalent to that of a quantum thermal machine, e.g. thermal accelerator, under the reverse-annealing schedule^58^. Here, we take a step further and analyze the energetic and computational performance of quantum annealing under reverse-annealing, and how to optimize it through the introduction of pausing in the annealing schedule. We demonstrate our approach on the D-Wave 2000*Q* quantum annealer and we show that a pause in the annealing schedule allows us to achieve better computational performance at a lower energetic cost. Additionally, we discuss the role and impact of the magnetic field on the performance of the chip.

## Theory & figures of merit

We start by briefly outlining notions and notations. Quantum annealing consists of mapping the optimization problem to a mathematical model, that can be described using qubits, i.e. the Ising model^59^. The quantum annealer is initialized in a quantum state that is easy to prepare. The system is then evolved according to a time-varying Hamiltonian, which is a mathematical operator representing the problem’s objective function, and can be expressed as,

1 $$\begin{aligned} H(s_t)=(1-s_t)\sum _i \sigma _i^x + s_t \left( \sum _i h_i \sigma _i^z + \sum _{\langle i,j \rangle } J_{i,j} \sigma _i^z \sigma _j^z \right) , \end{aligned}$$

where $\sigma _i^{\alpha }$, with $\alpha =(x,z)$ are Pauli matrices, and $h_i$ is the local magnetic field. $s_t=t/\tau$ describes the annealing schedule that controls the rate of the transformation between the easy-to-prepare Hamiltonian $H_0=\sum _i \sigma _i^x$ and the problem specific Hamiltonian $H_p= \sum _i h_i \sigma _i^z + \sum _{\langle i,j \rangle } J_{i,j} \sigma _i^z \sigma _j^z$, with $\tau \in [0,t]$. On the D-Wave machine, the annealing time *t* can be chosen from microseconds ($\sim \!2\mu s$) to milliseconds ($\sim \!2000 \mu s$).

The usual quantum annealing process, called forward annealing, starts with initializing the qubits in a known eigenstate of $\sigma ^x$. The system is then driven adiabitically by varying the Hamiltonian parameters, the adiabatic evolution is a requirement for the system to remain in its instantaneous ground state^60^. Initially, the driver Hamiltonian $H_0$ dominates, and the qubits are in a quantum superposition. As the annealing progresses, the problem Hamiltonian $H_p$ gradually becomes dominant, and the qubits tend to settle into the low-energy states that correspond to the optimal solution of the problem.

**Figure 1:**
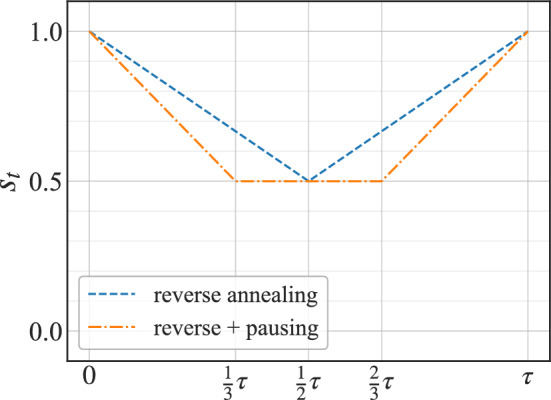
Quantum annealing protocols used in our demonstrations. $s_t$ is an annealing parameter, $s_t = t/\tau$, *t* represent time and $\tau$ is the total annealing time in $\mu$s.

In this work, we employ a different protocol called reverse-annealing as depicted in Fig. 1, where the processor initially starts with a classical solution defined by the user, to explore the local space around a known solution to find a better one. Reverse-annealing has been shown to be more effective than forward annealing in some specific use cases, including nonnegative/binary matrix factorization^61^, portfolio optimization problems^62^, and industrial applications^63^. Moreover, reverse-annealing has unique thermodynamic characteristics with typically enhanced dissipation^58,64^.

To quantify the thermodynamic efficiency of the D-Wave 2000*Q* chip, we initialize the quantum annealer in the spin configuration described by a thermal state at inverse temperature $\beta _1=1$, and we assume that initially the system (S)+environment (E) state is given by the joint density matrix,

2 $$\begin{aligned} \rho =\frac{\exp {(-\beta _1 H_S)}}{Z_S} \otimes \frac{\exp {(-\beta _2 H_E)}}{Z_E}, \end{aligned}$$

where $H_S$ and $H_E$ denote, respectively, the Hamiltonian of the system and environment, while $Z_S$ and $Z_E$ describe, respectively, the partition function of the system and environment. $\beta _2$ is the inverse temperature of the environment. The energy transfer between two quantum systems, initially at different temperatures, is described by the quantum exchange fluctuation theorem^65,66^,

3 $$\begin{aligned} \frac{p(\Delta E_1,\Delta E_2)}{p(-\Delta E_1,-\Delta E_2)}=e^{\beta _1 \Delta E_1 + \beta _2 \Delta E_2}, \end{aligned}$$

where $\Delta E_i, i=1,2$ are, respectively, the energy changes of the processor and its environment during the annealing time *t*, and $p(\Delta E_1,\Delta E_2)$ is the joint probability of observing them in a single run of the annealing schedule. Equation (3) can be re-written in terms on the entropy production $\Sigma =\beta _1 \Delta E_1 + \beta _2 \Delta E_2$ as^67,68^,

4 $$\begin{aligned} \frac{p(\Sigma ,\Delta E_1)}{p(-\Sigma ,-\Delta E_1)}=e^{\Sigma }. \end{aligned}$$

**Figure 2:**
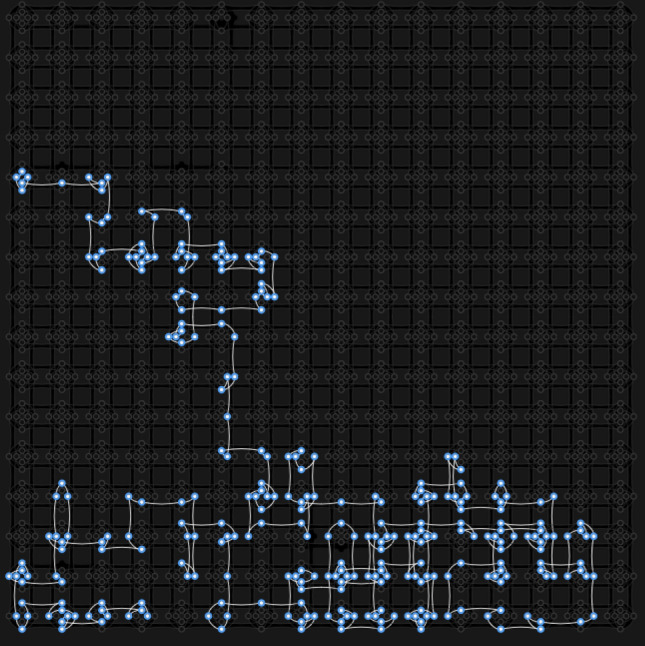
Example of the embedding of the $300-$spin Ising chain onto D-Wave 2000*Q* quantum processing unit (QPU) with the chimera architecture^69^. White-blue dots and lines are active qubits, and grey ones are inactive. This is one-to-one embedding, such that every physical qubit corresponds to one logical qubit.

Note that during our demonstrations, we have *only* access to the energy change of the processor $\Delta E_1$. Therefore, the thermodynamic quantities: entropy production $\langle \Sigma \rangle$, average work $\langle W\rangle$, and average heat $\langle Q\rangle$ are not directly accessible. However, they can be lower bounded by thermodynamic uncertainty relations^70^ as shown in Appendix B,

5 $$\begin{aligned} \langle \Sigma \rangle&\ge 2g\left( \frac{\langle \Delta E_1 \rangle }{\sqrt{\langle \Delta E_1^2 \rangle }}\right) , \end{aligned}$$

6 $$\begin{aligned} -\langle Q \rangle&\ge \frac{2}{\beta _2}g\left( \frac{\langle \Delta E_1 \rangle }{\sqrt{\langle \Delta E_1^2 \rangle }}\right) - \frac{\beta _1}{\beta _2} \langle \Delta E_1 \rangle , \end{aligned}$$

7 $$\begin{aligned} \langle W \rangle&\ge \frac{2}{\beta _2}g\left( \frac{\langle \Delta E_1 \rangle }{\sqrt{\langle \Delta E_1^2 \rangle }}\right) + \left( 1 - \frac{\beta _1}{\beta _2} \right) \langle \Delta E_1 \rangle , \end{aligned}$$

where $g(x)=x\tanh ^{-1}{(x)}$, and $\beta _2$ is the inverse temperature of the environment which can be estimated using the pseudo-likelihood method introduced in^71^. The factors influencing the tightness of the lower bounds, Eqs. (5), (7), are measurement errors and calibration issues, which we minimize by considering a large number of annealing runs (10000). Accordingly, the upper bound on the thermodynamic efficiency can be determined from

8 $$\begin{aligned} \eta _{\text {th}} \le - \frac{ \langle W \rangle }{ \langle Q \rangle }, \end{aligned}$$

which is not upper bounded by the Carnot efficiency that is defined for heat engines, as quantum annealers under reverse-annealing protocols behave like thermal accelerators^58^. Moreover, we are interested in analyzing the computational efficiency of the quantum annealer, which we define as

9 $$\begin{aligned} \eta _{\text {comp}} \le \frac{ {\mathscr {P}}_\text {GS}}{ \langle W \rangle }, \end{aligned}$$

and where have introduced the probability that ground state $s^\star$ is found in the given annealing run,

10 $$\begin{aligned} {\mathscr {P}}_{\text {GS}} = {\mathbb {P}}(s^\star \in {{\textbf {s}}}). \end{aligned}$$

This quantity is computed by dividing the number of successful runs (i.e., those which have found the ground state) by the total number of runs. We emphasize here that the definition Eq. (9), is not a universal metric for computational performance. It is specifically tailored to capture the unique computational characteristics of quantum annealers, which operate based on principles akin to the thermodynamic cycle of a thermal accelerator. The definition, Eq. (9), provides a contextual tool to evaluate the performance of quantum annealers in terms of energy consumption relative to computational output. Hence the dimension of 1/energy. Since information is physical, expressing computational efficiency in terms of energy provides a direct link to the physical process occurring during quantum annealing. The efficiencies defined by Eqs. (8) and (9) are the main figures of merit of our analysis.

It is also instructive to analyze the ratio of the theoretical ($E_{\text {th}}=-299$) ground state energy and the value read from the machine ($E_{\text {exp}}$),

11 $$\begin{aligned} Q_{\text {GS}} = \Big \langle \frac{E_{\text {exp}}}{E_{th}} \Big \rangle , \end{aligned}$$

which is averaged over the number of samples. The quality, Eq. (11), measures the accuracy between the experimentally obtained ground state energy from the quantum annealer and its theoretically predicted counterpart, normalized over the number of samples.

## Results and discussion

All our demonstrations were performed on a D-Wave 2000Q quantum annealer. The physical properties of this machine are presented in Table 1 of Appendix A^72^. We considered an antiferromagnetic (i.e. $\forall i \, J_{i, i+1} = 1$) Ising chain on $N\!=\!300$ spins, with Hamiltonian as defined in (1). However, one must first embed the given problem into the target quantum processing unit (QPU) architecture to perform quantum annealing. Here, embedding means finding a mapping between physical qubits presented in the machine and logical qubits (i.e. $\sigma ^z_i$) representing our problem. Figure 2 shows an example embedding of this Ising problem onto the QPU with Chimera architecture.

We used annealing schedules shown in Fig. 1. The system was initialized for both schedules by taking a sample thermal state at $\beta =1$ using Gibbs sampling^73^. For reverse annealing, with a given annealing time $\tau$, we ran $s_t \rightarrow 1/2$ for $t \in (0, \tau /2)$ and $s_t \rightarrow 1$ for the remaining time. In reverse annealing with pausing $s_t \rightarrow 1/2$ for $t \in (0, \tau /3)$, next for $t \in (\tau /3, 2\tau /3)$ $s_t = 1/2$, as we pause the annealing process. Lastly, for $t \in (2\tau /3, 1)$ we let $s_t \rightarrow 1$.

### Zero magnetic field – “naked performance”

We start with the case where the magnetic field *h* is turned off.

#### Reverse-annealing without pausing

**Figure 3:**
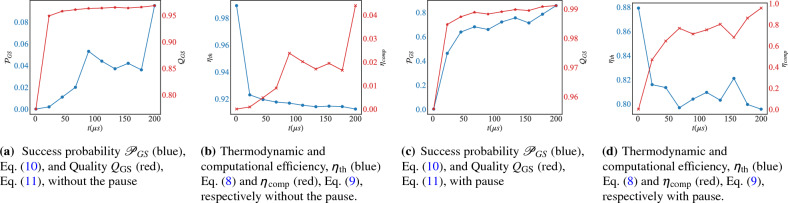
Figures of merit under reverse-annealing with respect to the total anneal time *t*. (a+b) without the pause and (c+d) with pause for $N=300$ spins, ferromagnetic couplings *J*, and zero magnetic field. Each point is averaged over 1000 annealing runs with 10 samples each.

Figure 3a,b report data from reverse-annealing demonstrations without the introduction of the pause. The success probability, Eq. (10), is shown in panel (a) where we see that under reverse-annealing without the pause, the success probability of finding the ground state energy is very low and does not exceed 10% in the whole annealing time. This means that in all 10000 samples taken in the demonstration, only less than 1000 samples provided the ground state energy. However, although the probability to reach the ground state energy is low, its quality, Eq. (11), which is shown in the same panel is very high and saturates at 0.95.

The results shown in panel (a) are consistent with the thermodynamic and computational efficiency, Eqs. (8) and  (9) respectively, reported in panel (b). We see that under reverse-annealing without the pause, the computational efficiency of the chip $\eta _{comp}$ grows with the annealing time *t* while being very low and not exceeding 4% in the whole annealing time, which follows the behavior of the success probability ${\mathscr {P}}_{GS}$. Energetically, the thermodynamic efficiency $\eta _{th}$ decays exponentially with $\tau$ and remains at a high value close to 1.

#### Reverse-annealing with pausing

The low computational performance of the chip for the simple Hamiltonian, Eq. (1), shows that the reverse-annealing protocol does not exploit the energetics of the chip efficiently. Ideally, one aims at finding the protocol that provides a high computational efficiency at the lowest possible thermodynamical cost. For this reason, we introduce a pause in the reverse-annealing protocol, as depicted in Fig. 1. Introducing a pause in the annealing schedule in quantum annealing has been shown to offer several benefits, such as: enhancing the probability of finding better solutions by efficient exploration of the solution space, which allows for a broader range of potential solutions to a given problem^74,75^. Furthermore, since the pausing duration can be manipulated by the user, this offers the ability to balance between exploration and exploitation, which allow for the fine-tuning of the solution quality. The flexibility offered by the pausing strategy also allows for adaptation to the characteristics of specific problem instances, which enhances the efficiency and effectiveness of quantum annealing for a wide range of applications^76^.

Figure 3c,d presents the results of applying a pause during the reverse-annealing schedule, as shown in Fig. 1. The success probability ${\mathscr {P}}_{GS}$ improves dramatically as shown in panel (c), where it grows quickly to 0.8 during the annealing schedule, which means that 80% of the 1000 annealing runs taken during the demonstration returned the ground state energy. The quality, Eq. (11), shown in the same panel also benefits from introducing the pause, where the overlap between the theoretical value and the energy read from the machine is almost unity. The power of pausing is even more significant for the thermodynamic and computational efficiency, Eqs. (8) and  (9) respectively, reported in panel (d). We see that pausing allows for achieving high computational efficiency at a moderate thermodynamic cost, which is due to the concept of thermalization. Introducing a pause in the annealing schedule allows the chip to relax and thermalize after being excited by quantum or thermal effects near the minimum gap. However, pausing is not always beneficial, and it depends on several factors such as the relaxation rate, the pause duration, and the annealing schedule. The optimal protocol corresponds to a pause right after crossing the minimum gap and its duration should be no less than the thermalization time^75^.

### Magnetically assisted annealing

**Figure 4:**
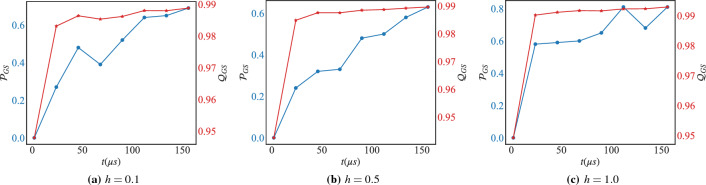
The success probability ${\mathscr {P}}_{GS}$ (blue), Eq. (10), and the Quality $Q_{\text {GS}}$ (red), Eq. (11), under reverse-annealing without the pause for different values of the magnetic field *h* and with respect to the total anneal time *t*. Each point is averaged over 100 annealing runs with 10 samples each.

**Figure 5:**
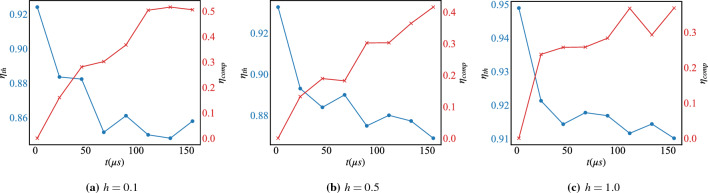
The thermodynamic and computational efficiency, $\eta _{th}$ (blue) Eq. (8) and $\eta _{comp}$ (red) Eq. (9) respectively, under reverse-annealing without the pause for different values of the magnetic field *h* and with respect to the total anneal time *t*. Each point is averaged over 100 annealing runs with 10 samples each.

**Figure 6:**
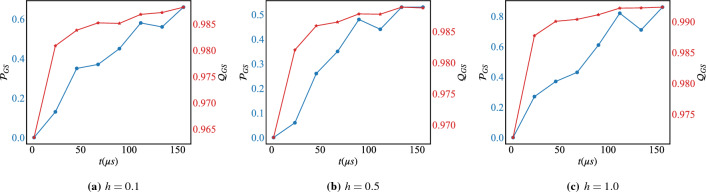
The success probability ${\mathscr {P}}_{GS}$ (blue), Eq. (10), and the Quality $Q_{\text {GS}}$ (red), Eq. (11), under reverse-annealing with the pause for different values of the magnetic field *h* and with respect to the total anneal time *t*. Each point is averaged over 100 annealing runs with 10 samples each.

**Figure 7:**
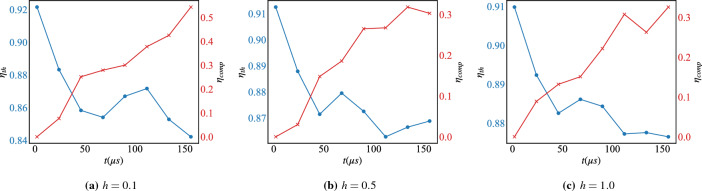
The thermodynamic and computational efficiency, $\eta _{th}$ (blue) Eq. (8) and $\eta _{comp}$ (red) Eq. (9) respectively, under reverse-annealing with the pause for different values of the magnetic field *h* and with respect to the total anneal time *t*. Each point is averaged over 100 annealing runs with 10 samples each..

Next, we perform demonstrations with the magnetic field switched on, under reverse-annealing with and without pausing. The local magnetic field plays a crucial role in shaping the energy landscape and controlling the behavior of the qubits during the annealing process. By manipulating the local magnetic field, quantum annealers can explore and optimize complex problem spaces more effectively.

#### Assisted reverse-annealing without pausing

The benefit of introducing the magnetic field is clear from the behavior of the success probability ${\mathscr {P}}_{GS}$, Eq. (10), reported in Fig. 4. In this case, without introducing a pause in the annealing schedule, the success probability of the ground state of the problem Hamiltonian is very high compared to the case when the magnetic field is off (c.f. Fig. 3a). Introducing the magnetic field influences the shape of the energy landscape that the qubits explore during the annealing process^77^. The landscape can be adjusted to promote or discourage certain configurations of the qubits, which can help guide the system toward the desired solution, and explains the slight improvement in the quality, Eq. (11), reported in the same panel. This dramatic improvement reflects itself on the thermodynamic and computational efficiency Eqs. (8) and (9) respectively, of the chip as reported in Fig. 5. In this case, introducing the magnetic field allows to guide the system in the energy landscape which is a more efficient strategy to exploit energy to perform computation.

##### Assisted reverse-annealing with pausing

Interestingly, in comparison with the case for $h\!=\!0$ reported in Fig. 3c,d, introducing a pause in the annealing schedule with the magnetic field being present decreases the success probability ${\mathscr {P}}_{GS}$, Eq. (10), as can be seen from Fig. 6. Consequently, it decreases also the thermodynamic and computational efficiency, $\eta _{th}$ Eq. (8) and $\eta _{comp}$ Eq. (9) respectively, as can be seen from Fig. 7. Switching on the magnetic field in quantum annealing changes the qubit energy levels, and structure. On the other hand, for pausing to work it needs to be carefully applied while taking into account the energy level structure variation with the magnetic field. For this reason, the pause needs to be performed right after the minimum gap characterized by the value of the magnetic field *h*^74,75^.

## Concluding remarks

We have investigated the optimization of the computational efficiency and the thermodynamic cost in the D-Wave quantum annealing systems employing reverse-annealing. By combining reverse-annealing with pausing, we have demonstrated improved computational efficiency while operating at a lower thermodynamic cost compared to reverse-annealing alone. Our results highlight the potential benefits of strategically incorporating pausing into the annealing process to enhance overall computational and energetic performance. Furthermore, our results indicate that the magnetic field plays a crucial role in enhancing computational efficiency during reverse-annealing. However, when pausing is involved, the magnetic field becomes detrimental to the overall performance. This suggests the need for careful consideration of the magnetic field configuration and its impact on the energy gap of the system during the annealing process.

While our demonstrations were performed on the D-Wave Chimera architecture, it will be interesting to extend our approach to the Pegasus and Zephyr architectures. These two models offer high tolerance to noise and a more complex structure, which allows us to investigate the trade-off between energetic performance and computational complexity. Additionally, exploring the scalability of these findings to larger-scale quantum systems and real-world applications remains a promising avenue for future research.

## Figures and Tables

**Figure 8:**
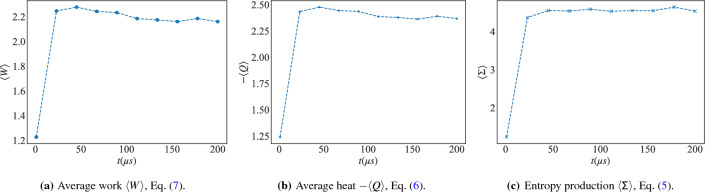
Figures of merit, Eqs. (5), (6), versus the total annealing time *t* under reverse-annealing with zero magnetic field.

**Figure 9:**
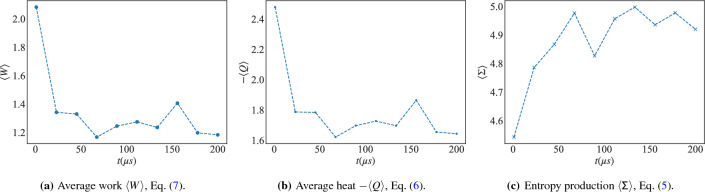
Figures of merit, Eqs. (5), (6), versus the total annealing time *t* under reverse-annealing and pausing with zero magnetic field.

**Figure 10:**
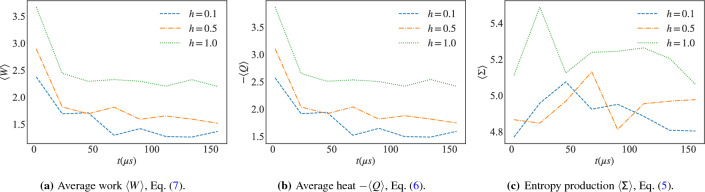
Figures of merit versus the total annealing time *t* under reverse-annealing with non-zero magnetic field.

**Figure 11:**
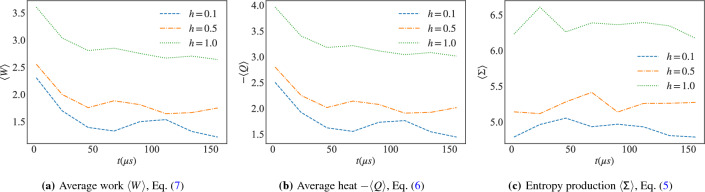
Figures of merit versus the total annealing time *t* under reverse-annealing and pausing with non-zero magnetic field.

## Data Availability

The code of the demonstrations is publically available in the Github repository^78^.

## Appendix A: Physical properties of the D-Wave 2000Q

We report in this appendix the physical properties of the 2000Q chip^72^

**Table 1:** Physical properties of the D-Wave 2000*Q* machine.

Parameter	Value
Qubits	2041
Couplers	5974
Qubit temperature (mK)	$13.5 \pm 1.0$
$\text {M}_{\text {AFM}}$ (pH)	1.795
Programming time ($\mu s$)	8767.90
Default post programming thermalization ($\mu s$)	1000.0
Average single qubit thermal width (Ising units)	0.103
Single qubit freezeout (scaled time)	0.719
Readout time ($\mu s$)	349.44
Readout error rate	0.0
Default post readout thermalization ($\mu s$)	0.0
Annealing slope range ($\mu s^{-1}$)	$-1.0$–1.0
Maximum anneal schedule points	12

## Appendix B: Derivation of lower bounds

The thermodynamic uncertainty relation (TUR) relates the amount of energy a system dissipates to the uncertainty of a certain observable in that system. For a process described by a joint probability distribution $p(\sigma ,\phi )$, the fluctuation relation is given by:

12 $$\begin{aligned} \frac{p(\sigma ,\phi )}{p(-\sigma ,-\phi )} = e^{\sigma }, \end{aligned}$$

where $p(\sigma ,\phi )$ is the probability of observing simultenously $\sigma$ and $\phi$ during the forward process, while $p(-\sigma ,-\phi )$ describe the same probablity but during the backward process. Then applying TUR gives^70^:

13 $$\begin{aligned} \langle \phi \rangle ^2 \le \langle \phi ^2 \rangle f(h^{-1}(\langle \sigma \rangle )), \end{aligned}$$

with $f(x)=\tanh ^2{(x/2)}$, and $h^{-1}=(x\tanh {(x/2)})^{-1}$. The bound on $\langle \sigma \rangle$ follows by manipulating Eq. (13):

14 $$\begin{aligned} \langle \sigma \rangle \ge 2g\left( \frac{ \langle \phi \rangle }{\sqrt{ \langle \phi ^2 \rangle }}\right) , \end{aligned}$$

with $g(x)=x\tanh ^{-1}(x)$. Applying Eq. (14) on the fluctuation relation Eq. (4) gives:

15 $$\begin{aligned} \langle \Sigma \rangle \ge 2g\left( \frac{\langle \Delta E_1 \rangle }{\sqrt{\langle \Delta E_1^2 \rangle }}\right) . \end{aligned}$$

Making use of $\Sigma =\beta _1 \Delta E_1 + \beta _2 \Delta E_2$ and $\langle W \rangle = \Delta E_1 + \Delta E_2$ yields to

16 $$\begin{aligned} -\langle Q \rangle&\ge \frac{2}{\beta _2}g\left( \frac{\langle \Delta E_1 \rangle }{\sqrt{\langle \Delta E_1^2 \rangle }}\right) - \frac{\beta _1}{\beta _2} \langle \Delta E_1 \rangle ,\end{aligned}$$

17 $$\begin{aligned} \langle W \rangle&\ge \frac{2}{\beta _2}g\left( \frac{\langle \Delta E_1 \rangle }{\sqrt{\langle \Delta E_1^2 \rangle }}\right) + \left( 1 - \frac{\beta _1}{\beta _2} \right) \langle \Delta E_1 \rangle . \end{aligned}$$

Figures 8, 9, 10, 11 report the behavior of the average work $\langle W\rangle$, heat $\langle Q\rangle$, and the entropy production $\langle \Sigma \rangle$, Eqs. (5), (6), under the annealing protocols in Fig. 1.
